# Fetal femur length and risk of diabetes in adolescence: a prospective cohort study

**DOI:** 10.1186/s41182-024-00611-6

**Published:** 2024-07-01

**Authors:** Urme Binte Sayeed, Evana Akhtar, Anjan Kumar Roy, Sharmin Akter, Ondine S. von Ehrenstein, Rubhana Raqib, Yukiko Wagatsuma

**Affiliations:** 1 Graduate School of Comprehensive Human Sciences, University of Tsukuba, 1-1-1 Tennodai, Tsukuba, Ibaraki 305-8575 Japan; 2 Nutrition Research Division, icddr, b, Dhaka, 1212 Bangladesh; 3 Departments of Community Health Sciences and Epidemiology, Fielding School of Public Health, University of California, Los Angeles, CA USA; 4 Faculty of Medicine, Department of Clinical Trials and Clinical Epidemiology, University of Tsukuba, Tsukuba, Ibaraki 805-3575 Japan

**Keywords:** Fetal femur length, Prediabetic biomarkers, Preadolescence, Adolescence, Matlab, Bangladesh

## Abstract

**Background:**

Diabetes is more apparent in adulthood but may be dormant in childhood and originates during early fetal development. In fetal biometry, femur length (FL) is crucial for assessing fetal growth and development. This study aimed to assess potential associations between fetal femur growth and prediabetic biomarkers in Bangladeshi children.

**Methods:**

A cohort study embedded in a population-based maternal food and micronutrient supplementation (MINIMat) trial was conducted in Matlab, Bangladesh. The children in the cohort were followed up until 15 years of age. In the original trial, pregnancy was confirmed by ultrasound before 13 gestational weeks (GWs). Afterward, ultrasound assessments were performed at 14, 19, and 30 GWs. FL was measured from one end to the other, capturing a complete femoral image. The FL was standardized by GW, and a *z*-score was calculated. FBG and HbA1c levels were determined in plasma and whole blood, and the triglyceride–glucose index, a biomarker of insulin resistance, was calculated as Ln [fasting triglycerides (mg/dl) × fasting glucose (mg/dl)/2]. Multivariable linear regression analysis using a generalized linear model was performed to estimate the effects of FL at 14, 19 and 30 GWs on prediabetic biomarkers at 9 and 15 years of age. Maternal micronutrient and food supplementation group, parity, child sex, and BMI at 9 years or 15 years were included as covariates.

**Results:**

A total of 1.2% (6/515) of the participants had impaired fasting glucose during preadolescence, which increased to 3.5% (15/433) during adolescence. At 9 years, 6.3% (32/508) of the participants had elevated HbA1c%, which increased to 28% (120/431) at 15 years. Additionally, the TyG index increased from 9.5% (49/515) (during preadolescence) to 13% (56/433) (during adolescence). A one standard deviation decrease in FL at 14 and 19 GWs was associated with increased FBG (*β* = − 0.44 [− 0.88, − 0.004], *P* = 0.048; *β* = − 0.59 [− 1.12, − 0.05], *P* = 0.031) and HbA1c (*β* = − 0.01; [− 0.03, -0.005], *P* = 0.007; *β* = − 0.01 [− 0.03, − 0.003], *P* = 0.018) levels at 15 years. FL was not associated with diabetic biomarkers at 9 years.

**Conclusion:**

Mid-trimester impaired femur growth may be associated with elevated prediabetic biomarkers in Bangladeshi adolescents.

## Background

Diabetes mellitus (DM) is one of the major non-communicable diseases worldwide and is associated with increased morbidity and mortality at all stages of life. According to the International Diabetes Federation (IDF), approximately 573 million people aged between 20 and 70 years are currently living with diabetes, which might increase to more than 700 million by 2045 [1]. In Bangladesh, about 13 million people were diagnosed as diabetic in 2021, with 5.7 million undiagnosed cases who were unaware of their metabolic disorder [2]. In addition, the increase in childhood-onset diabetes in Bangladesh is concerning. A retrospective study conducted using the clinical records of the diabetic association of Bangladesh revealed that from 2011 to 2018, in total, 725 children and adolescents (< 20 years old) were diagnosed diabetic, with a 12% average annual increase in the incidence of type 2 diabetes [3].

Type 2 diabetes mellitus (T2DM) might have an early onset in childhood, and in most cases, it remains asymptomatic and unrecognized [4]. Therefore, it is highly challenging to estimate the risk of diabetes in childhood. To date, studies have focused on lifestyle behaviors such as unhealthy diet, physical inactivity, sedentary behaviors, obesity, and rapid urbanization as risk factors for the development of T2DM in children and adolescents [5]. However, T2DM is a complex metabolic disorder, and it has been suggested that restricted fetal growth due to an adverse intrauterine environment may impact the risk of cardiometabolic diseases in adulthood [6]. The “Thrifty Phenotype” hypothesis was first introduced by Barker and Hales, which stated that fetal development is critical in human life and can influence the likelihood of developing non-communicable diseases later in life. It suggested that in an adverse intrauterine environment, the majority of nutrients in a fetal body are directed towards the vital organs, such as the brain and heart, essential for fetal survival, resulting in an undersupply of nutrition to the peripheral organs. Therefore, the imbalanced nutrient distribution can disrupt glucose homeostasis in the liver, muscles, and bones, potentially leading to insulin resistance [7]. Subsequently, the Developmental Origins of Health and Disease (DOHaD) concept was formulated and has gained wide acceptance in recent decades. This theory demonstrated that any threats during the preconception or prenatal periods, for instance, placental insufficiency or maternal undernutrition, can significantly impact the health of the offspring in adulthood [8]. According to DOHaD theory, a fetus experiencing undernutrition in utero may maintain good health later if the nutritional status remains consistent postnatally. Nevertheless, the children who confronted fetal growth restriction (FGR) due to suboptimal nutritional status are at a high risk of developing metabolic syndrome when exposed to excessive energy or an obesogenic diet after birth [9].

The GUSTO cohort study conducted in Singapore uncovered that postnatal catch-up growth from birth to age 2, irrespective of whether FGR had occurred, was associated with high cardiometabolic risks during childhood [10]. An animal study also revealed that mice experiencing rapid growth after intrauterine growth restriction (IUGR) showed greater insulin resistance than those with lower growth post IUGR [11]. A small-scale Australian cohort study reported that constrained fetal growth trajectories during early pregnancy were related to insulin resistance among young adults [12]. Furthermore, another cohort study demonstrated an inverse relationship between fetal biparietal diameter, birthweight, and insulin resistance among 20-year-old adults [13]. Additionally, a cohort in China showed that the fetuses exposed to a famine exhibited a 1.2-fold increased risk of developing type 2 diabetes in adulthood [14].

During pregnancy, fetal growth is assessed through various parameters, including head size measurements, abdominal circumference, and femur length (FL) [15]. FL is the sole bone measurement included in fetal growth assessments, providing accurate information about gestational age, fetal growth, and development [16]. The femur bone begins developing early in pregnancy but undergoes substantial growth during the second trimester, becoming apparent in ultrasound assessments [17]. As the largest bone in human body, the femur plays a significant role in high bone mineral deposition, including calcium, phosphorus, and magnesium [18]. Research suggests that these minerals, particularly calcium, are required for the optimal function of pancreatic β-cells [19]. Adequate calcium levels were reported to be correlated with enhanced insulin sensitivity, facilitating efficient cellular response to insulin and glucose uptake [20]. In addition, recent studies indicate that bones and skeletal muscles have endocrine functions, particularly in regulating glucose metabolism. Osteocalcin, a bone-derived protein, has been shown to be significantly associated with increasing insulin sensitivity and glucose metabolism [21]. Therefore, an impaired fetal femur growth could impact glucose metabolism in adulthood through reduced nutrition and mineral deposition.

The risk of IGT, and therefore, the risk of T2DM, is more evident in adulthood, yet such metabolic dysfunction may originate during early fetal development [22]. Most studies have focused on the effect of overall growth patterns resulting in low birth weight on the development of T2DM later in life [23, 24]. Nevertheless, birth weight does not provide information about the longitudinal fetal growth pattern. Also, the estimated fetal weight (EFW) is used for predicting fetal growth and potential neonatal or maternal complications during and after delivery [25]. However, EFW is a combined measure of multiple fetal parameters that can be affected by various external factors [26]. Consequently, it does not indicate specific fetal growth and development. In this study, we aimed to understand whether the growth pattern of the femur bone during fetal stage influences the risk of metabolic disorders later in life.

## Methods

### Study design, area, and participants

**Fig. 1:**
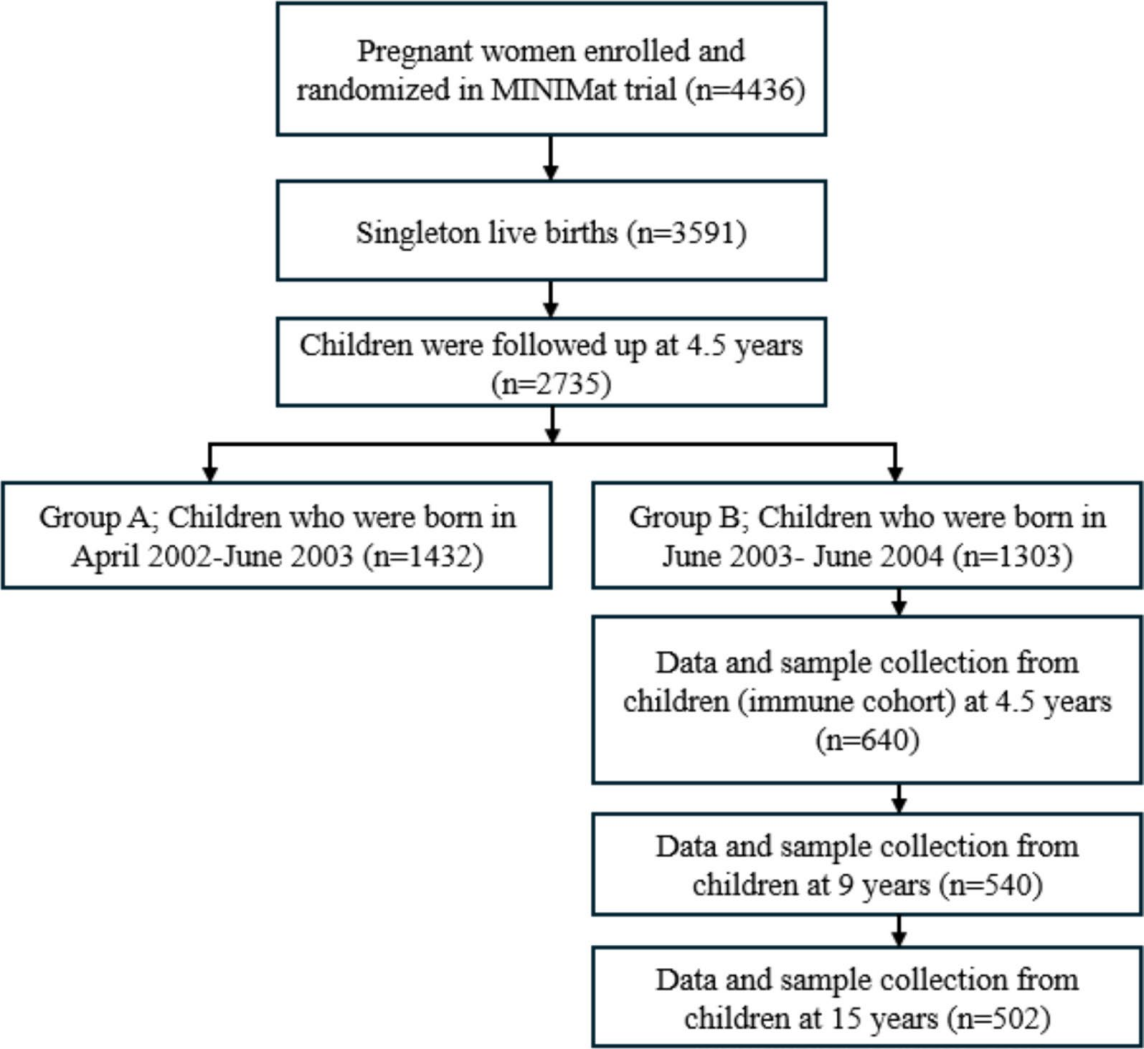
Flowchart of children recruitment and metabolic biomarkers analyzed in a smaller cohort nested in the MINIMat trial

This was a cohort study embedded in a population-based food and micronutrient supplementation trial (MINIMat trial) conducted between 2001 and 2003 in Matlab, a rural area of Bangladesh (ISRCTN 16581394), to examine the efficacy of several food and multimicronutrient supplements during pregnancy to improve birth and neonatal health outcomes among 4436 pregnant women. The primary outcomes of the MINIMat trial were the possible effects of prenatal food and micronutrient enrichment on maternal weight gain, hemoglobin status and infant mortality [27]. Matlab is approximately 75 km southeast of Dhaka (the capital of Bangladesh), where the International Centre for Diarrheal Disease Research, Bangladesh (ICDDR, B), a global health research institute, runs a health and demographic monitoring system with four connected health care centers offering health care to the local population, covering 246,893 people [28]. In the trial, women were confirmed to be pregnant by the community health research staff during monthly scheduled house visits based on the last menstrual period (LMP) and a urine test. Pregnant women, preferably at 8 to 13 weeks of gestation were invited to the nearest health care center for pregnancy confirmation through ultrasound and, therefore, to be enrolled in the study. The recruited participants were randomly assigned to two food groups, early (gestational week 9) or usual (gestational week 20) and subdivided into three micronutrient groups from gestational week 14: (a) 30 mg iron with 400 mg folic acid; (b) 60 mg iron with 400 mg folic acid; and (c) 15 different micronutrient preparations by UNICEF. Maternal anthropometric measurements, gestational age at birth, and infant mortality were also recorded in the MINIMat trial. During the pregnancy, about 20% of women were lost to follow-up due to spontaneous abortion (*n* = 236), migration (*n* = 188), stillbirth (*n* = 89), withdrawal of consent (*n* = 129), and others and approximately 1.8% of the live-born infants were twins (*n* = 65) [27]. During the antenatal ultrasound assessments, the cases of congenital malformations, e.g., spina bifida and hydrocephalus, were referred to tertiary hospitals in Dhaka city. Therefore, they were lost to follow-up and excluded from the study. In total, 3591 women gave birth to singleton live-born infants. The children were then examined at 4.5 years, 9 years, and 15 years of age [29]. To minimize the number of tests and the repetitive collection of biological samples from each child, the children were divided into two groups after 4.5 years of age based on their birth years (Group A: April 2002–June 2003; Group B: June 2003–June 2004) [30]. A sub-cohort of children born primarily in facilities from Group B was selected and referred to as the ‘Immune cohort’ (*n* = 640). We evaluated the same group of children derived from the immune cohort at 9 years as ‘preadolescents’ and 15 years as ‘adolescents’. About 16% of children from the immune cohort were lost to follow-up before 9 years (*n* = 540) because of migration (*n* = 42), withdrawal of consent (*n* = 39), refusal of blood collection (*n* = 7) and others. Finally, at 15 years old, we followed up with 502 children as 38 participants were lost to follow-up due to migration (Fig. 1).

### Fetal growth markers and birth anthropometry

All the participants in the MINIMat trial were assessed using ultrasound during pregnancy. The first ultrasound test was performed during enrollment (between 8 and 13 weeks of gestation) to determine the gestational age (GA). Enrolled participants were invited for further ultrasound exams at nearly 14 weeks, 19 weeks, and 30 weeks of gestation. During each ultrasound test, fetal biometry parameters were measured. FL was measured by capturing a complete femoral picture from end to end. Three measurements were taken at each session, and the average of these measurements was calculated [30]. This study used fetal femur length measurement as the predictor variable. Fetal FL is a crucial fetal biometry parameter for assessing potential growth restriction [31]. The femur is the longest bone in the human body, with substantial bone mineral deposition. Thus, short fetal femur length might induce alteration in glucose metabolism, therefore, metabolic risks later in life. Fetal growth measurements were taken using an ultrasound machine with a 3.5 MHz standard convex probe (SSA 320A, Justavision-200; Toshiba, Tokyo, Japan). In total, four ultrasound machines were installed at four clinics (one ultrasound machine at each clinic) [30]. The ultrasound measurements were performed by nine trained paramedics following the WHO ultrasound guidelines [32]. Detailed information regarding the inter- and intra-observer variance in ultrasound measurements is provided elsewhere [33]. The fetal parameters were standardized by gestational age. Expected means and standard deviations were calculated using Chitty’s formula [34]. Subsequently, *z*-scores for the fetal parameters were derived from the observed average mean, expected mean, and standard deviation. The mothers’ recalled LMP was used to calculate the number of gestational weeks. Missing LMP data were replaced with ultrasound determined LMP data. A change in fetal femur length was described by a shift in FL *z*-scores.

In 2002–2004, about 40% of all children were born in healthcare facilities and for the infants delivered at home, a birth notification system was administered, assisting paramedics to measure the birth anthropometry. Birth weight was measured primarily within 72 h of delivery using SECA electronic scales (SECA, Hamburg, Germany) with an accuracy of 10 g. Birth weights measured within the first 24 h post-delivery were not adjusted. However, the measurements collected from 24 h to 30 days after birth were adjusted using a standard deviation score (SDS) transformation based on the assumption that infants maintained the same relative position within the anthropometric distribution during this period [35, 36].

### Diabetic biomarkers

Blood samples were collected from the participants at both 9 (*n* = 540) and 15 (*n* = 460) years of age. Briefly, at the Matlab field site, blood samples were collected in lithium-heparin-containing tubes, and plasma was separated from the venous blood by centrifugation. Finally, both whole blood and plasma samples were sent to the ICDDR, B Dhaka lab and stored at − 80 °C. On the day of analysis, the frozen plasma samples were thawed, vortexed, and analyzed to determine fasting blood glucose and triglyceride (TG) levels by enzymatic colorimetry using the fully automated clinical chemistry analyzer Cobas c311 (Roche Diagnostics Mannheim, Germany). Glycated hemoglobin (HbA1c) in whole blood was measured by a turbidimetric inhibition immune assay using a Cobas c311 [29]. The triglyceride–glucose (TyG) index was computed as Ln [fasting TG (mg/dl) × fasting glucose (mg/dl)/2] [37]. The TyG index has emerged as a reliable surrogate marker for insulin resistance (IR). Research indicates that the TyG index is a superior biomarker for detecting type 2 diabetes mellitus, outperforming HOMA-IR, the most common diabetic biomarker in children and adults [38]. During childhood, determining a reference or cutoff value for indicating IR is challenging due to the pubertal stage and various underlying physiological changes, preventing the establishment of a standard reference value. Nevertheless, due to the simplicity and efficiency, studies have proved that TyG can effectively predict the risk of diabetes among these groups of people. Therefore, in this study, the TyG index has been considered a diabetic biomarker due to its effectiveness in identifying diabetes risk among children and adolescents [39, 40]. In this study, prediabetes was defined as FBG level of ≥ 100 mg/dL, HbA1c level of ≥ 5.7% to 6.4%, and TyG index of more than 8.6 [41, 42].

### Statistical analyses

Descriptive statistics were used to describe the study participants and the distribution of diabetic biomarkers. Bivariate analysis was carried out using Pearson correlation between the predictor variables (FL *z*-scores at gestational weeks 14, 19 and 30), outcome variables (diabetic biomarkers: FBG, HbA1c and the TyG index) and the covariates (mentioned below). All predictor and outcome variables were assessed for linearity in scatter plots. Multivariate linear regression analysis using a generalized linear model (linear function) was used to estimate associations between FL *z*-scores at 14, 19 and 30 gestational weeks and diabetic biomarkers at 9 and 15 years of age. A mediation analysis was performed using the Sobel test, assuming the adolescents’ BMI (at the 15-year model) might have a mediating effect on the outcomes. However, the adolescents’ BMI did not act as a mediator in the 15-year model. Hence, participants’ BMI at 9 and 15 years (at 9-year and 15-year models) were considered as covariates. The models were adjusted for the following covariates: maternal food (categorical; early and usual) and micronutrient supplements (categorical; 30 mg Fe with 400 mg folic acid, 60 mg Fe with 400 mg folic acid and 15 different micronutrient preparations by UNICEF) taken in early pregnancy (MINIMat trial), parity (ordinal), child sex (categorical; male and female), BMI (continuous) at 9 years of age (for the 9-year model) and BMI (continuous) at 15 years of age (for the 15-year model). The covariates were selected based on the known risk factors. Initially, maternal BMI at gestational weeks 8 was also considered as a potential confounder. However, the variable did not significantly influence the outcomesat both 9 and 15 years. Thus, the variable was excluded later from the models. Residual distributions were checked for all outcomes using residual and fitted plots. BMIs at 9 and 15 years of age were positively skewed. Therefore, the variables were Ln transformed to obtain normally distributed residuals in the regression analyses. Stratification analysis was performed for the FL *z*-score at gestational weeks 14, 19 and 30 with maternal food and supplementation groups during pregnancy for each outcome in both 9 and 15 years models. However, no significant differences in outcomes were found among the groups. Additionally, no effect modification of the FL *z*-score and child sex were observed. Thus, gender-stratification analysis was not performed. Furthermore, multicollinearity was verified among the independent variables for each outcome. We defined statistical significance as 5%. SPSS version 29.0 was used for all the analyses.

## Results

### Maternal and child characteristics

Out of 540 children at 9 years, we excluded 24 due to unavailable valid birth anthropometric data. Hence, 516 children were included in the 9-year analysis. Also, at 15 years, 69 children were excluded from the analysis because of no valid birth anthropometric data (*n* = 27) and refusal to blood collection (*n* = 42). Therefore, 433 children were included in the analysis. Table 1 shows the characteristics of the mothers and their children. The mean (SD) maternal age was 26.4 (5.8) years at the time of recruitment for the MINIMat trial. At baseline (gestational week 8), the mean (SD) maternal BMI was 20.6 kg/m^2^ (2.9). The ratio of male to female children was 1:1.04. At birth, 24% of the children had low birth weights (< 2500 g), and 10% were born preterm (< 37 weeks of gestation). The mean (SD) BMI of the children at 9 years of age was 14.3 kg/m^2^ (1.7), and that at 15 years of age was 19.1 kg/m^2^ (3.5). The median FL *z*-score was 0.4 (range: − 8, 11) at gestational week 14, 0.9 (range: − 7.0, 9.6) at gestational week 19 and 0.5 (range: − 4.8, 5.2) at gestational week 30.

**Table 1:** Maternal and children characteristics

Characteristics	Value
Maternal characteristics (*n* = 516)	
Maternal age (year); mean, SD	26.4 ± 5.8
Mothers’ BMI at gestational week 8; mean, SD	20.6 ± 2.9
Primigravida; *n* (%)	156 (30.4%)
Children characteristics (*n* = 516)	
Child sex; males; *n* (%)	253 (49%)
Low birthweight (< 2500 g); *n* (%)	123 (24%)
Preterm birth (< 37 gestational weeks); *n* (%)	50 (10%)
BMI at 9 years old; mean, SD	14.3 ± 1.7
BMI at 15 years old; mean, SD (*n* = 433)	19.1 ± 3.5
FLz score at gestational weeks 14; median, range	0.4 (− 8, 11)
FLz score at gestational weeks 19; median, range^a^	0.9 (− 7.0, 9.6)
FLz score at gestational weeks 30; median, range	0.5 (− 4.8, 5.2)

^a^Missing value (*n* = 10)

### Distribution of diabetic biomarkers at 9 and 15 years of age

Only 1.2% of the children had a higher FBG level at 9 years of age (100–125 mg/dl), which increased to 3.5% at 15 years of age. During preadolescence, 6.3% of the children had HbA1c level of 5.7% to 6.4%, while the percentage increased to 28% during adolescence. On the other hand, the TyG index indicated that 9.5% of the children were in the prediabetic stage (> 8.6) at 9 years of age, which was 13% at 15 years of age (Table 2).

**Table 2:** Diabetic biomarkers at 9 and 15 years of age of children living in Matlab

Metabolic biomarkers	Children (*n* = 516)	Boys (*n* = 254)	Girls (*n* = 262)
At 9 years old age			
Fasting blood glucose (FBG)^a^, mg/dl	77 ± 9.2	78 ± 9.6	76 ± 8.7
Glycated hemoglobin (HbA1c)^b^, %	5.2 ± 0.3	5.2 ± 0.3	5.2 ± 0.3
Triglyceride- glucose (TyG) index^c^	8 ± 0.4	8 ± 0.4	8.1 ± 0.4

Metabolic biomarkers	All (*n* = 433)	Boys (*n* = 205)	Girls (*n* = 228)
At 15 years of age (*n* = 433)			
Fasting blood glucose (FBG), mg/dl	78.2 ± 10.3	78.1 ± 11.6	78.3 ± 9
Glycated hemoglobin (HbA1c)^d^, %	5.5 ± 0.3	5.5 ± 0.2	5.5 ± 0.3
Triglyceride–glucose (TyG) index	8.1 ± 0.4	8 ± 0.5	8.1 ± 0.4

TyG index was calculated by Ln [fasting TG (mg/dl) × fasting glucose (mg/dl)/2] [37]

^a^Missing value (*n* = 1)

^b^Missing value (*n* = 8)

^c^Missing value (*n* = 1)

^d^Missing value (*n* = 2)

### Association of standardized fetal femur length with diabetic biomarkers during preadolescence

The FL z-score at gestational weeks 14, 19 and 30 was not associated with any diabetic biomarkers (FBG (at GW 14, *β* = − 0.08, 95% CI = − 0.43, 0.26; at GW 19, *β* = − 0.10, 95% CI = − 0.52, 0.32; at GW 30, *β* = − 0.18, 95% CI = − 0.79, 0.42), HbA1c (at GW 14, *β* = 0.01, 95% CI = − 0.001, 0.02; at GW 19, *β* = 0.01, 95% CI = − 0.003, 0.02; at GW 30, *β* = 0.01, 95% CI = − 0.007, 0.03), or TyG index values [at GW 14, *β* = − 0.006, 95% CI = − 0.02, 0.009; at GW 19, *β* = − 0.003, 95% CI = − 0.02, 0.01; at GW 30, *β* = − 0.01, 95% CI = − 0.04, 0.008)] at 9 years of age; only a few children were found to have any indication of positive diabetic markers at this age (Table 3).

**Table 3:** Association of FL_*z* score and diabetic biomarkers at 9 years old age

Biomarkers	Gestational weeks 14 (*n* = 516)		Gestational weeks 19 (*n* = 506)^a^		Gestational weeks 30 (*n* = 516)	
	*β* (95% CI)	*P*-value	*β* (95% CI)	*P*-value	*β* (95% CI)	*P*-value
FBG (mg/dl)^b^	− 0.08 (− 0.43, 0.26)	0.636	− 0.10 (− 0.52, 0.32)	0.641	− 0.18 (− 0.79, 0.42)	0.552
HbA1c (%)^c^	0.01 (− 0.001, 0.02)	0.087	0.01 (− 0.003, 0.02)	0.119	0.01 (− 0.007, 0.03)	0.208
TyG index^d^	− 0.006 (− 0.02, 0.009)	0.438	− 0.003 (− 0.02, 0.01)	0.718	− 0.01 (− 0.04, 0.008)	0.164

The model was adjusted for mothers’ micronutrient supplementation (categorical) and food supplementation (categorical) during pregnancy, parity (ordinal), children sex (categorical), BMI at 9 years (continuous). BMI at 9 years was ln transformed.

*β* regression coefficient, *CI* confidence interval, *TyG index* triglyceride–glucose index, *FBG* fasting blood glucose, *HbA1c* glycated hemoglobin

^a^Missing value (*n* = 10)

^b^Missing value (*n* = 1)

^c^Missing value (*n* = 8)

^d^Missing value (*n* = 1)

### Association of standardized fetal femur length with diabetic biomarkers during adolescence

Table 4 shows the estimated associations of the FL *z*-score with diabetic biomarkers at 15 years of age. The FL *z*-score at gestational weeks 14 and 19 was negatively associated with the FBG level at 15 years of age (*β* = − 0.44, 95% CI = − 0.88, − 0.004, *P* = 0.048) (*β* = − 0.59, 95% CI = − 1.12, − 0.05, *P* = 0.031). However, the FL *z*-score at gestational week 30 did not affect the FPG level at 15 years of age. A one-SD decrease in the FL *z*-score at gestational weeks 14 and 19 was significantly associated with a higher HbA1c level at 15 years of age (*β* = − 0.01, 95% CI = − 0.03, − 0.005, *P* = 0.007) (*β* = − 0.01, 95% CI = − 0.03, − 0.003, *P* = 0.018). Nevertheless, the FL *z*-score at gestational week 30 was not associated with the HbA1c level during adolescence. Additionally, no association was found between any gestational week measurement and the TyG index in adolescents.

**Table 4:** Association of FL_*z* score and diabetic biomarkers at 15 years old age

Biomarkers	Gestational weeks 14 (*n* = 433)		Gestational weeks 19 (*n* = 424)^a^		Gestational weeks 30 (*n* = 433)	
	*β* (95% CI)	*P*-value	*β* (95% CI)	*P*-value	*β* (95% CI)	*P*-value
FBG (mg/dl)	− 0.44 (− 0.88, − 0.004)	0.048	− 0.59 (− 1.12, − 0.05)	0.031	− 0.49 (− 1.27, 0.28)	0.212
HbA1c^b^ (%)	− 0.01 (− 0.03, − 0.005)	0.007	− 0.01 (− 0.03, − 0.003)	0.018	− 0.01 (− 0.03, 0.008)	0.218
TyG index	− 0.01 (− 0.03, 0.001)	0.064	− 0.01 (− 0.03, 0.006)	0.153	− 0.01 (− 0.04, 0.01)	0.313

The model was adjusted for mothers’ micronutrient supplementation (categorical) and food supplementation during pregnancy (categorical), parity (ordinal), children sex (categorical), BMI at 15 years (continuous). BMI at 15 years was ln transformed

*β* regression coefficient, *CI* confidence interval, *TyG index* triglyceride–glucose index, *FBG* fasting blood glucose, *HbA1c* glycated hemoglobin

^a^Missing value (*n* = 9)

^b^Missing value (*n* = 2)

## Discussion

Our findings indicate that a shorter standardized fetal femur length at gestational weeks 14 and 19 was associated with higher fasting blood glucose and glycated hemoglobin levels among 15-year-old Bangladeshi adolescents which did not appear in their preadolescence.

Fetal growth restriction is a public health concern that is considered to be a predictor of developing cardiometabolic diseases in adulthood. As previously discussed, the “Thrifty Phenotype” hypothesis explains that nutritional stress during fetal development compromises the development of peripheral organs, leading individuals to poorer tolerance of various physiological and environmental changes later in life. The populations undergoing rapid industrialization, characterized by increased dietary fat and sugar intake and decreased physical activity levels, are initially exposed to inadequate glycemic control and eventually to diabetes [43]. Also, the DOHaD concept has advanced our understanding of how animals and humans respond to adverse environmental conditions by altering their characteristics to cope with adversity. However, studies are being conducted to identify a more integrated and longitudinal approach in the future to predict the risk of developing metabolic disorders at the earlier stage of life. As the primary application of the DOHaD concept involves identifying, thereby preventing, or mitigating the likelihood of developing diseases later in life, a stronger emphasis is needed to identify the risk factors before symptoms manifest in adolescence to reduce the disease burden in adulthood [44].

FGR diminishes the β-cell mass responsible for insulin secretion and increases peripheral glucose and insulin sensitivity, which leads to impaired glucose tolerance and the onset of T2DM later in life [45]. Furthermore, in animal-based studies, FGR due to placental insufficiency showed metabolic alterations and led to insulin resistance in skeletal muscles during early adulthood [46, 47]. Fetal femur length is an essential part of fetal ultrasonography, which can predict potential restricted fetal growth during pregnancy [48]. Short fetal femur length mid-pregnancy has been proved to be associated with an increased risk of small for gestational age, preterm birth, and poor perinatal outcomes [49]. A cohort study in China demonstrated that fetuses with fast femur growth in early and mid-pregnancy had higher insulin levels in cord blood at birth [50].

The underlying pathway by which fetal femur bone length elevates diabetic biomarkers has not been explored. However, recent evidence in bone biology suggests that bone and skeletal muscles have endocrine functions, especially in glucose metabolism [51]. Osteocalcin, a bone-derived protein secreted from osteoblasts, has repeatedly been found to increase β-cell mass proliferation and improve insulin sensitivity and glucose homeostasis [52]. Studies have shown that serum osteocalcin is a multifunctional bone marker that is inversely associated with higher blood glucose levels, HbA1c levels and increased insulin resistance in adults [53–55]. The serum osteocalcin level has been reported to have an inverse relationship with the HbA1c level among diabetic patients, which indicates that osteocalcin might play a crucial role in glycemic control [56]. The femur is the largest bone in the human body and is responsible for high bone mineral deposition [57]. FGR might affect chondrocyte formation and function, reducing femur bone development [48]. Therefore, a short femur length might lead to reduced bone mineral deposition and lower osteocalcin levels in the fetus. The development of the human skeleton commences during fetal growth via endochondral bone formation, which starts with embryonic mesenchymal cells [58]. FGR was found to reduce mesenchymal proliferation and endochondral ossification, resulting in altered postnatal bone mineralization in animal models [59, 60]. Studies in both animals and humans have shown that maternal diet during the third trimester of pregnancy significantly influences bone mass density in offspring [61, 62]. Furthermore, a prospective cohort study showed that fetal and postnatal growth patterns affect bone mineral density in both SGA and normal-birth-weight infants [63].

Our study revealed that fetal femur length at gestational week 30 was not associated with elevated diabetes risk during adolescence. The potential reason might be the peak bone mineral deposition in the fetus around the last trimester. During fetal life, the placenta actively exchanges essential minerals, including calcium, phosphorus, and magnesium. Studies have proved that the accumulation of such minerals, especially calcium, increases by about 80% in the last trimester, which is essential for skeletal development and mineralization [64, 65].

Additionally, no association was found between fetal femur bone length and metabolic risk at 9 years of age. Metabolic changes occur as age increases. Most of the well-established consensus definitions and cutoff points for predicting the risk of metabolic syndrome are targeted for adults. However, it is challenging to identify the ideal cutoff values to predict metabolic syndrome in children aged < 10 years, as cardiometabolic risks might be hidden because of several physiological and pubertal changes [66]. Hence, although we did not detect any statistically significant associations between fetal femur length and diabetic biomarkers at 9 years of age, we detected a marked association between femur bone length and an increased risk of diabetes at 15 years of age among the same study sample.

This study has several strengths. To our knowledge, this is the first longitudinal cohort study examining the associations between fetal femur bone length and elevated diabetic biomarkers in Bangladeshi children. Further important strengths include the well-characterized cohort of pregnant women with an accurate gestation period and multiple available ultrasound measurements. Additionally, the participants were recruited over a full calendar year with multiple timepoints of data collection. Consequently, we were able to recruit and follow up most of the study participants who met the inclusion criteria.

## Limitation of the study

The limitations of the study included the lack of information on the family history of chronic diseases, which might have affected the metabolic risks among the adolescents. Also, this study included children who were primarily born in facilities, which might have influenced its generalizability. Additionally, we did not collect samples from the children to evaluate calcitonin or osteocalcin markers, which may be associated with fetal femur bone growth. Future studies explaining the underlying pathways by which fetal femur bone length influences glucose metabolism in adulthood are recommended.

## Conclusion

In conclusion, a shorter fetal femur length during mid-pregnancy may be associated with elevated prediabetic biomarkers among Bangladeshi adolescents. This study provides evidence indicating that reduced fetal femur length may increase the risk of prediabetes in adolescence. Adolescence is a crucial stage for the early onset of chronic diseases. Therefore, further research is imperative to explain how fetal growth restriction, irrespective of lifestyle modification, may contribute to the development of metabolic disorders in adulthood. Moreover, it is important to educate prospective parents about pre-pregnancy nutrition and practices that promote healthy fetal development to reduce the incidence of fetal growth restriction and low birth weight.

## Data Availability

The datasets relating to this study are available upon reasonable request.

## Abbreviations

DM: Diabetes mellitus
T2DM: Type 2 diabetes mellitus
FGR: Fetal growth restriction
IUGR: Intrauterine growth restriction
EFW: Estimated fetal weight
DOHaD: Developmental origin of health and diseases
SGA: Small for gestational age
GA: Gestational age
GW: Gestational weeks
FL: Femur length
BMI: Body mass index
TyG Index: Triglyceride-glucose Index
TG: Triglyceride
FBG: Fasting blood glucose
HbA1c: Glycated hemoglobin
IDF: International Diabetes Federation
IGT: Impaired glucose tolerance
IFG: Impaired fasting glucose
IR: Insulin resistance
ICDDR, B: International Centre for Diarrheal Disease Research, Bangladesh
LMP: Last menstrual period
WHO: World Health Organization
SD: Standard deviation
CI: Confidence interval
